# Intramuscular delivery of formulated RNA encoding six linked nanobodies is highly protective for exposures to three Botulinum neurotoxin serotypes

**DOI:** 10.1038/s41598-022-15876-2

**Published:** 2022-07-08

**Authors:** Jean Mukherjee, Celinia A. Ondeck, Jacqueline M. Tremblay, Jacob Archer, Michelle Debatis, Alexa Foss, Junya Awata, Jesse H. Erasmus, Patrick M. McNutt, Charles B. Shoemaker

**Affiliations:** 1 Department of Infectious Disease and Global Health, Cummings School of Veterinary Medicine, Tufts University, 200 Westboro Rd, North Grafton, MA 01536 USA; 2 Wake Forest Institute for Regenerative Medicine, Wake Forest School of Medicine, Winston-Salem, NC 27101 USA; 3 HDT Bio, Seattle, WA USA

**Keywords:** Biological techniques, Biotechnology, Drug discovery, Medical research

## Abstract

Single domain antibodies (sdAbs), also called nanobodies, have substantial biophysical advantages over conventional antibodies and are increasingly being employed as components of immunotherapeutic agents. One particularly favorable property is the ability to link different sdAbs into heteromultimers. This feature allows production of single molecules capable of simultaneously targeting more than one antigen. In addition, cooperative binding of multiple linked sdAbs to non-overlapping epitopes on the same target can produce synergistic improvements in target affinity, variant specificity, and in vivo potencies. Here we seek to test the option of increased component sdAbs in these heteromultimers by testing different sdAb heterohexamers in which each of the six camelid sdAb components (VHHs) can neutralize one of three different Botulinum neurotoxin (BoNT) serotypes, A, B or E. Each heterohexamer bound all three targeted BoNT serotypes and protected mice from at least 100 MIPLD_50_ of each serotype. To test the potential of mRNA therapeutics encoding long sdAb heteromultimers, one heterohexamer was encoded as replicating RNA (repRNA), formulated with a cationic nanocarrier, and delivered to mice via intramuscular injection. Heterohexamer antitoxin serum expression levels were easily detected by 8 h post-treatment, peaked at 5–10 nM around two days, and persisted for more than three days. Mice treated with the formulated repRNA one day post-treatment survived challenge with 100 MIPLD_50_ of each toxin serotype, demonstrating the function of all six component VHHs. Use of long sdAb multimers, administered as proteins or repRNA, offer the potential for substantially improved versatility in the development of antibody-based therapeutics.

## Introduction

Monoclonal antibody (mAb) immunotherapeutics are used for a broad spectrum of clinical applications. Recent efforts to improve mAb potencies and to expand their specificities have included the generation and testing of multifunctional antibody fusion proteins that recognize two^1^, three^2–4^ or more epitopes^5^. Such agents can offer synergistic improvements in binding to a single target or the addition of new functionalities through the binding and co-localization of multiple targets. A promising approach to next generation immunotherapeutics is to employ linked single-domain Abs (sdAbs) comprising the antigen binding domain of camelid^6^ or shark^7^ heavy-chain only Abs. These single domain nanobodies, which are referred to as VHHs, have distinct advantages over conventional antibodies (Abs) for clinical use, including their small size (~ 14 kDa), increased resistance to pH and temperature extremes, and inexpensive production in microbial hosts^8–10^. VHHs are also well-expressed as multimeric proteins, which can increase potency and enable the recognition of multiple therapeutic targets^11^. Finally, VHH heteromultimers are well suited to rapid expression in vivo when encoded in repRNA^12^.

One proven application of VHH multimers is as therapeutics against biological toxins. Protein toxins are important virulence factors in many important diseases and can cause harm through accidental or deliberate exposure. Currently, immunoglobulin-based antitoxins are the primary treatment modality for toxin exposures. Traditionally, these antitoxins are produced in agricultural species and passively administered as antiserum or purified Abs. However, traditional antitoxins are often expensive, difficult to access, and pose safety concerns due to species-specific anaphylaxis or cross-reaction with human proteins. Use of humanized mAb antitoxins reduces the risk of anaphylaxis but is currently limited by treatment costs and shelf-life, which make widespread stockpiling impractical. Furthermore, many toxin threats are found in nature with distinct sequence variations, requiring pools of mAbs for efficacy against all natural variants. VHH multimers could overcome these limitations through their multispecificity, lower manufacturing cost, rapid optimization in vitro and improved stability. To date, most reports of VHH antitoxins use genetically linked heterodimer proteins to increase antitoxin potency compared to pools of the same two monomer VHHs^13–15^. Some reports have successfully employed VHH heterotrimer and heterotetramer antitoxins^16–20^, thus further expanding the potential applications of VHH heteromultimers. In this report, we test the option of even longer heteromultimers by producing fully functional VHH heterohexamers containing six linked VHHs; three pairs of VHHs that each potently neutralize a different BoNT serotype.

The use of formulated RNA encoding therapeutic or immunogenic proteins is gaining promise in treating and preventing disease, most notably as vaccines for the prevention of COVID-19. We previously reported that intravenous delivery of lipid nanoparticle (LNP)-formulated mRNA encoding a VHH heterodimer protects mice from a high challenge dose of BoNT/A^12^. An important parameter of antitoxin efficacy is the ‘treatment window’, i.e., the time post-exposure during which an antitoxin can successfully protect a patient from a toxin challenge. In mouse models, there was no significant difference in treatment window when we compared treatments with antitoxin VHH proteins to intravenous treatments with LNP-formulated mRNA^12^. These data suggest RNA therapy is a promising approach for the rapid development and delivery of new botulinum antitoxins. However, intravenous administration of LNP-formulated mRNA limits therapeutic use to hospital settings. Alternatively, we recently demonstrated that intramuscular delivery using a replicating RNA (repRNA) enhances immunoglobulin expression up to 32-fold compared to non-replicating mRNA^21^. In contrast to conventional mRNA which simply encodes the gene-of-interest, repRNA encodes the replication machinery of an alphavirus, including the nonstructural protein genes and the non-coding RNA regulatory elements required for replication of that RNA and further transcription of a subgenomic RNA encoding the gene-of-interest^22^. In doing so, we can take advantage of virus evolution that has selected for enhanced protein expression from subgenomic RNA in the presence of host innate immune responses. One practical advantage of this approach is a dose-sparing effect essential for enabling therapeutic expression levels from a limited number of transfected muscle cells in vivo*.* Here we tested whether intramuscular delivery of LNP-formulated repRNA encoding anti-botulinum VHH multimers protects against lethal BoNT challenge.

Using mouse models of BoNT intoxication, we show that intravenous administration of VHH heterohexamer proteins or intramuscular administration of formulated repRNA encoding a VHH heterohexamer, confers broad-spectrum protection against supralethal doses of BoNT serotypes A, B and E. Through combining the speed of VHH development against novel threat agents with the versatility of RNA therapy, we demonstrate a potentially transformative method for administering broad-spectrum countermeasures against multiple pathogen threats.

## Results

### Expression of VHH heterohexamer proteins

**Figure 1:**
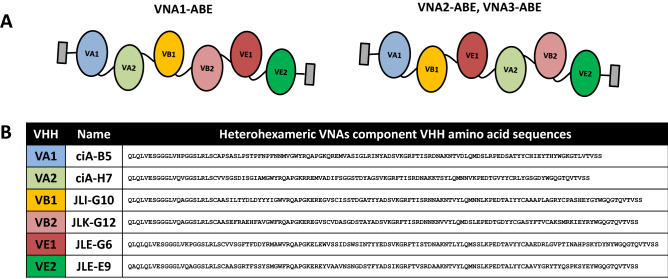
Design and expression of heterohexameric VNA antitoxins. (**A**) Cartoon image showing the VHH component structures of VNA1-ABE and VNA2-ABE. The six VHHs are flanked by E-tag peptides (gray box) for detection. (**B**) The lab names and amino acid sequences of the six component VHHs; VA1, VA2, VB1, VB2, VE1, and VE2. The original VHH sequences are available on GenBank (see Availability of data and materials).

Two synthetic genes, each encoding six linked VHH components, were designed. The resulting heterohexamer VHH-based neutralizing agents, called VNAs, contain pairs of VHHs against each BoNT serotype (A, B and E) assembled in two different configurations (Fig. 1A). The precise binding sites and mechanisms of BoNT neutralization have been characterized in structural and functional studies for each VHH. The VHHs used in this study include ciA-H7 (VA1) and ciA-B5 (VA2), which neutralize BoNT/A^15,23^; JLI-G10 (VB1) and JLK-G12 (VB2), which neutralize BoNT/B^23^; and JLE-G6 (VE1) and JLE-E9 (VE2), which neutralize BoNT/E^24^. In the heterohexamer VNA1-ABE, VHHs are linked together sequentially such that VHHs recognizing each BoNT serotype are in apposition, e.g., NH_2_- VA1/VA2/VB1/VB2/VE1/VE2. VNA1-ABE was assembled using flexible spacers to allow VHH pairs to bind two non-overlapping epitopes on their target proteins^15,23,24^. In VNA2-ABE, VHHs are linked in series, e.g., NH_2_- VA1/VB1/VE1/VA2/VB2/VE2. VNA2-ABE contains short SGGGG spacers between each VHH to reduce the total protein size. Both VNA1-ABE and VNA2-ABE coding regions are flanked by E-tags for detection and contain a C-terminal mouse albumin binding peptide to promote longer serum VNA half-life^25^. The component monomer VHH sequences are provided in Fig. 1B and complete heterohexamer VNA sequences are provided in Figure S1A.

**Figure 2:**
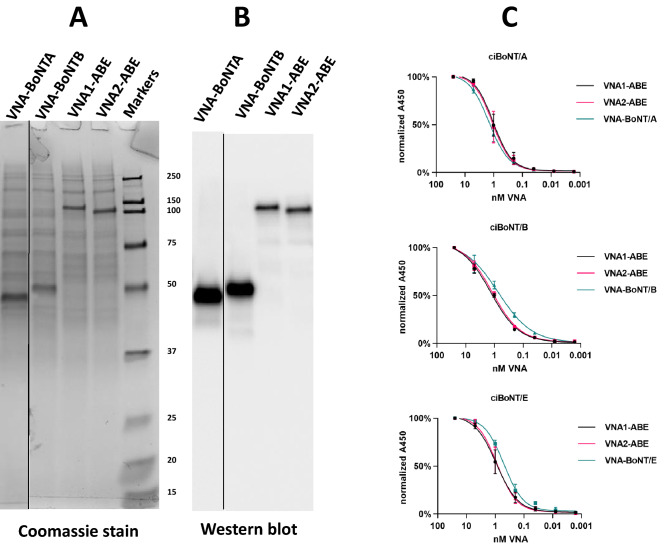
SDS-PAGE, western blot and VNA ELISA analysis of conditioned medium obtained from CHO cells transfected with expression vectors encoding the different VNAs. Conditioned media samples obtained as described in Materials and Methods were loaded onto SDS-PAGE for Coomassie staining (**A**) or western blotting (**B**). Western blots were probed with HRP/anti-E-tag, molecular weight marker sizes are shown in kDa. The full-size original stained gel and western blot are shown in Figure S3. (**C**) Dilution ELISAs of the various VNA conditioned media were performed with Costar tissue culture 96-well plates coated with 1 µg/ml of ciBoNTA, ciBoNTB or ciBoNTE. VNAs in conditioned media were quantified by comparison of staining intensity to protein standards. VNA-BoNTE was expressed in bacteria, purified and quantified as previously described^24^. All VNAs were initially diluted to 25 nM and then serially diluted 1:5. VNA binding was detected with HRP/anti-E-tag reagent. None of the LC-inactivating mutations in the ciBoNTs are located near the component VHH binding sites or affect VHH binding. Note that the EC_50_ values determined from dilution ELISAs likely underrepresent the true affinities of these agents for their native BoNT targets because the ciBoNT targets, which do not have an epitope tag for antibody capture, were thus coated directly onto ELISA plates. Studies have shown that VHH components, for which binding is typically conformation-dependent, have lower affinity for BoNTs absorbed to plastic vs antibody-capture^24^.

To confirm the neutralizing capabilities of each VNA, synthetic DNAs encoding VNA1-ABE and VNA2-ABE were first ligated in frame to the human Igκ leader coding sequence in the mammalian cell expression vector, pSecB. Similar pSecB vectors were prepared containing VNA heterodimers of BoNT/A neutralizing VHHs VA1/VA2 (VNA-BoNTA) and BoNT/B-neutralizing VHHs, VB1/VB2 (VNA-BoNTB). Each plasmid was transfected into CHO cells grown in serum-free medium and conditioned medium was collected after 3–4 days. Conditioned media were separated by SDS-PAGE followed by total protein staining or immunoblot analysis to confirm expression of full-length VNA heterohexamers (Fig. 2A,B). The secreted E-tagged VNA products, which were the major protein species in each conditioned medium, were of the expected sizes and their identities were confirmed by the E-tag blots. Truncated expression products were not detected. The expression level of each heterohexamer VNA was approximately 10 µg/ml, based on comparisons of the staining intensity against protein standards. Similar protein expression levels were obtained in conditioned media from cells transfected with the two heterodimer VNA vectors (Fig. 2A). Finally, a BoNT/E-neutralizing VNA heterodimer (VNA-BoNTE) was expressed in bacteria and purified by affinity chromatography as previously described^24^.

### VHH heterohexamers recognize their cognate BoNT targets

To determine whether assembly of six VHHs into heterohexameric VNAs had compromised target recognition as compared to heterodimer VNAs, dilution series ELISAs were performed to BoNT/A, /B and /E. To reduce the risk to laboratory workers, we used catalytically inactive BoNT variants (named ciBoNTA, ciBoNTB and ciBoNTE), in which the BoNT light chains were genetically inactivated^26,27^. Each heterohexamer VNA recognized all three toxin proteins with nearly identical apparent affinities (Fig. 2C, Table S1). Heterodimer VNAs exhibited EC_50_ values that were similar to VNA1-ABE and VNA2-ABE for binding to BoNT/A and /B. Similarly, the BoNT/E heterodimer VNA bound BoNT/E with an apparent affinity similar to VNA1-ABE and VNA2-ABE. These results demonstrate that VNA1-ABE and VNA2-ABE have affinities similar for BoNT/A, /B and /E to their respective VHH heterodimers. Thus, expressing six VHHs as a single protein did not compromise binding affinities compared to the heterodimer VHH pairs, nor was there an apparent difference between the two heterohexamer configurations.

### Recombinant VHH heterohexamers protect mice from intoxication by their three BoNT targets

**Figure 3:**
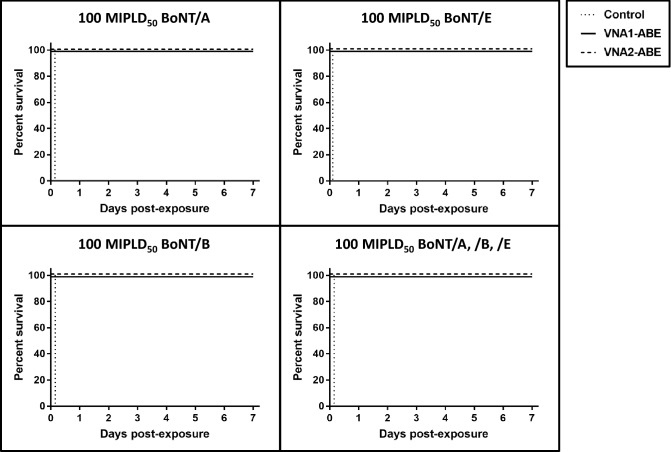
Heterohexamer VNAs protect mice from 100 MIPLD_50_ doses of BoNT/A, BoNT/B and BoNT/E. Mice (n = 5 per group) were co-administered 10 pmol VNA1-ABE or VNA2-ABE and 100 MIPLD_50_ of BoNT/A, BoNT/B or BoNT/E, or a combination of all three toxins, as indicated. Mice were monitored for survival over 7 d. In all cases, VNA treatment significantly increased survival rates from 0 to 100% (*p* = 0.008 versus vehicle for all VNA treatments; Fisher exact test).

We next performed in vivo mouse studies to assess the antitoxin efficacies of the CHO-expressed heterohexamer proteins. Mice were challenged with 100 mouse intraperitoneal median lethal doses (MIPLD_50_) (mouse intraperitoneal median lethal dose) of BoNT/A, 100 MIPLD50 of BoNT/B, 100 MIPLD50 BoNT/E, or a cocktail containing 100 MIPLD_50_ each of BoNT serotypes A, B and E (totaling 300 MIPLD_50_) and treated with vehicle, VNA1-ABE or VNA2-ABE (10 pmol each; n = 5 each group; Fig. 3). Whereas vehicle-treated mice died within 3 h of toxin administration, all mice given VNA1-ABE or VNA2-ABE survived through seven days (*p* = 0.008 for each group versus vehicle, Fisher exact test). While treated mice developed transient mild signs of botulism, all symptoms fully resolved within a week (not shown). These data are consistent with previous studies showing the cooperative binding conferred by VNAs improves antitoxin protection in comparison to the same VHHs given as monomers^13–15^. This was specifically shown to be the case for the VA1 and VA2 VHH monomer components of the heterohexamer, which were previously demonstrated to be completely ineffective as a pool when co-administered with 100 MIPLD_50_ of BoNT/A^15^. Likewise, pooled delivery of VE1 and VE2 VHHs provided no protection to mice co-administered > 2.5 MIPLD_50_ of BoNT/E^24^, whereas here they are highly protective when linked together. The protective effects provided by heterohexamer treatments to each BoNT serotype illustrates that the linked VHH components retained most or all toxin binding and neutralizing activities in vivo.

**Figure 4:**
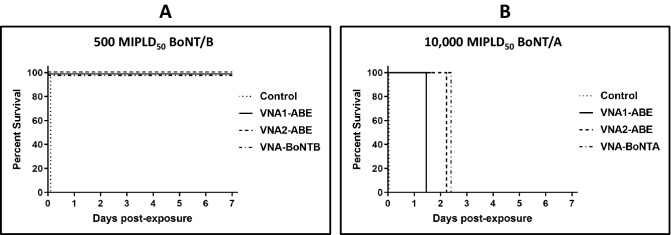
Heterohexamer VNAs protect mice from 500 MIPLD_50_ doses of BoNT/B and delay death from 10,000 MIPLD_50_ doses of BoNT/A. (**A**) Mice were co-administered 500 MIPLD_50_ of BoNT/B plus either saline vehicle (n = 5), 20 pmol VNA1-ABE (n = 5) or 20 pmol VNA2-ABE (n = 5) and monitored for survival over 7 d. Treatment with VNA1-ABE or VNA2-ABE increased survival versus vehicle (*p* = 0.0002 each; Fisher exact test). (**B**) Kaplan–Meier survival curves of mice co-administered 10,000 MIPLD_50_ of BoNT/B plus either saline vehicle (n = 5), 10 pmol VNA1-ABE (n = 5) or 10 pmol VNA2-ABE (n = 5). Among all groups, VNA treatment significantly increased MTD (χ^2^ = 20.7; *p* < 0.0001, Mantel-Cox log-rank test). In pairwise comparisons, treatment with VNA1-ABE or VNA2-ABE increased MTD versus vehicle (*p* = 0.0002 each) and VNA2-ABE-treatment increased MTD compared to VNA1-ABE-treatment (*p* = 0.003).

We performed two additional mouse challenge studies to determine whether the heterohexamer VNA configuration affected results. In one study, mice were challenged with a 500 MIPLD_50_ dose BoNT/B and we compared groups treated with either the heterohexamer VNA1-ABE or VNA2-ABE (Fig. 4A). Vehicle control mice died within two hours while mice treated with each VNA showed 100% survival (*p* = 0.0002 vs vehicle, Fisher exact test). In a second study, mice were challenged with 10,000 MIPLD_50_ of BoNT/A and treated with vehicle, VNA1-ABE or VNA2-ABE (Fig. 4B). While vehicle-treated mice died within 1 h after challenge, consistent with such a high toxin dose, all VNA treatments increased median time-to-death (MTD) versus vehicle (*p* = 0.0016 each, Kaplan–Meier log-rank test). Moreover, VNA2-ABE-treatment increased MTD compared to VNA1-ABE (2.25 d vs 1.5 d; *p* = 0.003), suggesting that the increased spacing between cognate VHH pairs in VNA2-ABE enhanced their neutralization capacity versus VNA-ABE1 (Figs. 1 and S1). Collectively, these data confirmed heterohexamer VNAs retain the protective efficacies previously reported for their corresponding heterodimer VNAs.

### Formulated repRNA encoding a VHH heterohexamer antitoxin protects mice from intoxication by all three BoNT serotype targets

Having characterized the in vivo efficacies of pre-expressed VNAs against supralethal toxin challenge doses, we next sought to test whether intramuscular injection of a formulated repRNA encoding an optimized VNA heterohexamer similarly prevented botulism toxemia in vivo. For RNA delivery, we generated a new heterohexamer VNA (VNA3-ABE, Figure S1). VNA3-ABE used the VHH order from VNA2-ABE based on the improved MTD observed in Fig. 4B. To reduce the total size of the heterohexamer protein, VNA3-ABE was further modified to: (1) remove non-core VHH residues, (2) retain a single C-terminal E-tag for detection, and (3) reduce spacer length between VHHs by replacing them with five glycine residue spacers. VNA3-ABE DNA was synthesized, incorporated into a repRNA vector (RNA/VNA3-ABE) and formulated with a cationic nanocarrier, termed LION™ (HDT Bio) for intramuscular delivery. LION is a newly described cationic nanocarrier which can be stably stockpiled for over a year at 4 C and mixed with an RNA of choice immediately before use^21^. Such a workflow is ideal for antitoxin applications where antitoxin RNA can be selected from a library and simply mixed with the stockpiled formulation, precluding the need to co-manufacture a custom formulation for each type of intoxication.

**Figure 5:**
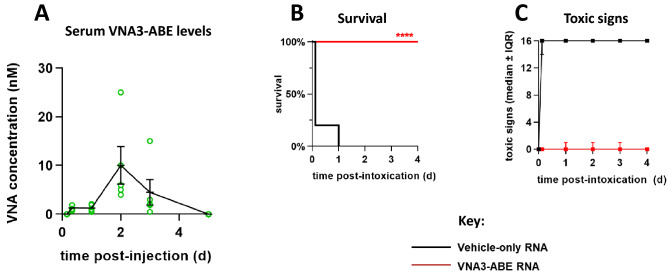
Intramuscular treatment with LION-formulated repRNA encoding VNA3-ABE protects mice from 100 MIPLD_50_ doses of BoNT/A, BoNT/B and BoNT/E. (**A**) ELISA quantitation of VNA3-ABE serum levels from 4 h to 5 d after RNA/VNA3-ABE administration (n = 5 per time point). Naïve serum was used as an assay control. (**B**) Kaplan–Meier survival curves following treatment with LION vehicle (n = 10) or RNA/VNA3-ABE (n = 10) and intoxication with 100 MIPLD50 each of BoNT/A, /B and /E. Survival curves were compared using Mantel-Cox log-rank test (χ^2^ = 21.2; *p* < 0.0001). (**C**) Progression of toxic signs in vehicle- and RNA/VNA3-ABE-treated mice.

We first performed a pharmacokinetic (PK) study to assess RNA/VNA serum expression levels as a function of time post-administration. Mice (n = 30) were given 10 µg of RNA/VNA3-ABE, prepared in LION, and delivered by bilateral injection into the thigh muscle (5 µg per muscle). Terminal bleeds were performed between 4 h and 5 d after injection (n = 4 mice per time point) and sera were assessed for VNA3-ABE protein levels by quantitative dilution ELISAs (Figure S2). VNA3-ABE was readily detectable by 8 h, peaked at 2 d, and declined below the limit of detection by 5 d (Fig. 5A). On a molar basis, VNA3-ABE serum protein levels at 8 h (~ 1 nM) far exceeds peak BoNT serum levels after an intravenous 1 LD_50_ challenge (55 fM)^28^, suggesting RNA-formulated VNA3-ABE could result in protective levels of antitoxin between 4 and 8 h after intramuscular delivery.

We next tested the therapeutic benefits of RNA/VNA3-ABE in mouse BoNT challenge studies. LION-formulated RNA/VNA3-ABE (10 ug per mouse) or LION alone was administered via bilateral thigh injections. At 24 h post-injection, mice were challenged by intravenous injection with a cocktail containing 100 MIPLD_50_ of BoNT/A, /B and /E (cumulative 300 MIPLD_50_; n = 10 mice per group). All control mice died within 3 h after BoNT intoxication, consistent with short survival times following high-dose challenges (Fig. 5B)^23^. In comparison, all mice receiving RNA/VNA3-ABE survived challenge (*p* < 0.0001 vs LION alone). Despite the substantial challenge dose, VNA-ABE treated mice exhibited only minor signs of botulism, which rapidly resolved (Fig. 5C). Collectively, these data demonstrated formulated RNA encoding VNA3-ABE protected against lethal challenge with multiple BoNT serotypes.

## Discussion

In this report, we exploited our large panel of BoNT-binding single-domain antibody VHHs to design and engineer three heterohexamer VNAs, each containing the same six BoNT-neutralizing VHHs. The VNAs differed in the order and/or the spacing of the six VHHs. We show that each VNA can protect mice from high-dose challenge with all three of the BoNT serotypes responsible for most human botulism. The heterohexamer VNAs were effective whether administered intravenously as proteins (VNA1-ABE, VNA2-ABE) or administered intramuscularly as formulated repRNA (VNA3-ABE). The smallest heterohexamer successfully tested has a secreted MW of only ~ 84 kDa. The high efficacy of the VNAs in protecting mice from 100 MIPLD_50_ challenges for all three BoNT serotypes, as compared to unlinked monomer pools, demonstrates that each linked component VHH retained most or all of its inherent toxin binding and neutralizing properties within this multifunctional heteromultimer.

The three heteromultimeric VNAs were designed with the same VHH components distributed in either a VA1-VA2-VB1-VB2-VE1-VE2 (amino to carboxy terminal) series (VNA1-ABE) or a VA1-VB1-VE1-VA2-VB2-VE2 series (VNA2-ABE, VNA3-ABE). We speculated that the VA1-VB1-VE1-VA2-VB2-VE2 series might offer greater flexibility in how the protein bound each serotype. In this regard, we have shown that VHH heterodimer antitoxin potency can be substantially improved when VHHs are separated by spacers of sufficient size to permit two VHHs to bind simultaneously to the same toxin molecule^23^. Because both heterohexameric VNAs provided similar high potencies against each toxin, no major difference in the two designs were revealed in this study. It is likely that the bulky, tightly-folded nature of each VHH component did not offer sufficient spacing or structural flexibility to permit simultaneous binding of two VHH component to each toxin to detectably improve potency. Future structural studies may permit predictions as to the contributions of intervening VHHs to the spacing between different component VHHs within long multimers, and thus facilitate the future design of heteromultimeric VNAs with enhanced potencies.

The recent major technical advances in the manufacture of formulated mRNA, and the remarkable safety and clinical success of the mRNA COVID-19 vaccines, has highlighted the potential of formulated mRNA as a viable and desirable route of delivery for both vaccines and immunotherapeutics. A recent report employing formulated mRNA to deliver antitumor, antiviral and antitoxin antibodies, including VHH heterodimers, in animal models was shown to be capable of eliciting therapeutically effective serum antibody levels^12^. This study also demonstrated that RNA delivery of a heterodimeric VNA neutralizing BoNT/A was as effective as the protein VNA in preventing botulism in mice when administered intravenously at different times post-intoxication. However, this approach utilized intravenous administration of RNA which would be largely limited to use in hospital settings. Clearly the availability of intramuscular (IM) delivery would offer greater future clinical utility of RNA administered immunotherapeutics, particularly for antitoxins so as to facilitate a more rapid response to a bioterror event.

To test whether a complex heterohexameric VNA could be effectively administered via the more practical IM route, we utilized repRNA for its self-amplifying characteristics that contribute to improved expression efficiency^29^. Specifically we employed repRNA formulated in lipid inorganic nanoparticle (LION) which has been shown to result in improved serum levels of antibodies following IM administration^21,30^ For this test, we designed a VNA in the VA1-VB1-VE1-VA2-VB2-VE2 series that encoded only the minimal ‘core’ of each VHH based on structural data. Crystal structures show that the alpaca VHH functional core structure begins at the consensus QVQLVE amino end of framework 1 (FR1) and ends at the consensus VSS carboxyl terminus in framework 4 (FR4)^23^. We also employed a minimal spacer encoding 5 glycine residues between each VHH component. Only a single epitope tag peptide (E-tag) was included for detection. One advantage of this design is to minimize the size of the encoded protein. A second advantage is to minimize the potential for VNA immunogenicity, since the core VHHs themselves are very similar to human V_H_ domain and are poorly immunogenic in human clinical trials^31,32^. In the future, any immunogenicity could be further reduced through humanization of the VHH components^33^.

Our studies showed that IM delivery of the LION-formulated repRNA produced easily detectable serum VNA levels after 8 h. Peak serum levels of 5–25 nM occurred at 2 days despite the known short intrinsic half-life of VNAs (~ 1 h)^25^. Serum VNA levels became undetectable at day 5. These PK data were similar to prior studies treating mice with an mRNA encoded VNA administered in an intravenous LNP formulation^12^, although peak levels were lower and more variable as expected due to intramuscular administration. The latter observation is more pronounced in smaller animals likely due to a requirement for accurate intramuscular delivery for efficient uptake of repRNA as, in other studies, we have observed lack of uptake when LION/repRNA is delivered subcutaneously. Nonetheless, serum VNA levels observed in these mice were anticipated to be in substantial molar excess to serum toxin levels after administration of 100 MIPLD_50_ challenge doses. This prediction was validated as the LION-formulated repRNA treatments was fully protective in mice when administered 1 day prior to the 100 MIPLD_50_ challenge doses for all three BoNT serotypes.

There are several options to improve treatments with RNA-encoded heteromultimeric VNAs. Given the successes of mRNA vaccines against SARS-CoV-2, we anticipate the chemistry of RNA formulations will be further optimized to improve speed, amplitude and duration of protein expression. For post-exposure treatments, the latency to effective VNA serum levels will be critical and thus reducing latency will be a key research objective. If the immunogenicity of core VHH components proves to be an issue, additional efforts will focus on developing humanized VHH components or new formulations that minimize nanoparticle delivery to antigen-presenting cells. Perhaps the most significant modification would be to improve VNA serum levels and PK properties. One approach to improve PK properties is to include an antibody Fc domain at the carboxyl end of the VNAs. Appending an Fc domain is known to substantially extend the serum half-lives of recombinant peptides and proteins^34^, thereby providing greater and longer lasting therapeutic benefit. Addition of Fc domains also increases serum antibody levels as the fusion proteins accumulate in serum for a longer time before being cleared. As an alternative to Fc domains, incorporation of an additional VHH component that binds to abundant, long-lived serum proteins such as albumin has also been reported to improve PK properties^35^.

In summary, we have demonstrated that VHH heteromultimers with six component VHHs can be efficiently expressed and secreted in mammalian cells while retaining the function of each VHH component. We also demonstrate that a single VHH heterohexamer can be produced that effectively protects animals from intoxication by three different toxins, in this case BoNT/A, BoNT/B and BoNT/E. This work highlights the great potential of VHH antibodies to retain high their individual functionality even when expressed as multifunctional chains of linked VHHs containing at least six VHH components.

## Materials and methods

### Ethics statement

In vivo studies were performed were performed in accordance with the relevant guidelines and regulations established by the Guide for the Care and Use of Laboratory Animals of the National Institutes of Health and were approved by the Institutional Animal Care and Use Committees (IACUCs) of Tufts University (Protocol G2016-74) and Wake Forest School of Medicine (Protocol A20-162). This study is reported in accordance with ARRIVE guidelines.

### Toxins

BoNT/A, BoNT/B and BoNT/E complex preparations (subtype 1 for each toxin, all from Metabiologics Inc.) were used for in vivo assays. Prior to use, BoNT/E was subjected to trypsin activation^28^. Briefly, 25 µL of 1.2 mg/ml trypsin (EMD #650,211) in HEPES (Sigma #H0887), 50 µL of 1 mg/ml complexed BoNT/E and 100 µL of sterile PBS were incubated 30 min. at 37 °C. Following incubation, 25 µL of 2.5 mg/ml trypsin inhibitor (Sigma-Aldrich #T6522) was added and the mixture further incubated 15 min. at room temperature. The resulting solution containing 0.25 ug/ul of activated BoNT/E was stored at − 80 °C until use. The median lethal dose (MLD50) for each toxin preparation was calculated using the standard mouse lethality assay as previously described^36,37^. BoNT/A, BoNT/B and activated BoNT/E were diluted to working concentrations in PBS with 0.2% gelatin (Sigma-Aldrich #501,785,182) immediately before use and administered to mice via intraperitoneal (500 µL total volume) injection.

### VHH and VNA expression

For expression of most VNAs, a CHO cell host was employed. DNA encoding the VNAs was synthesized by Genscript with restriction sites compatible with insertion in frame into a mammalian expression vector based on pSecTag2. The sequences of encoded heterohexamer proteins are shown in Figure S1. Similar expression vectors were engineered for expression of heterodimeric VNAs encoding VHHs VA1/VA2 (VNA-BoNTA) or VB1/VB2 (VNA-BoNTB). Expression of the heterodimeric VNA-BoNTE containing VE1/VE2 is described as Trx/E/JLE-G6/JLE-E9/E in^24^ and was produced and purified from *E. coli*. VNAs contained E-tags for detection. The mammalian expression plasmids were transfected into CHO-S cells in suspension cultures of serum-free FreeStyle™ CHO cell medium. Cells were transfected using FreeStyle™ MAX reagent (Invitrogen) according to the manufacturer’s recommendations and incubated under orbital shaking for three days. Conditioned medium was harvested under sterile conditions and stored at 4 °C. VHH and VNA expression levels were estimated by comparison to protein standards using BioRad Image Lab software. VNA-BoNTE was expressed in E. coli as previously reported^24^.

### ELISAs

ELISAs were performed using Costar tissue culture 96 well plates to reduce the conformational deformation that occurs with conventional ELISA plastic^24^. Catalytically inactive BoNT proteins representing BoNT/A, BoNT/B and BoNT/E^26,27,38^ (kindly provided by Dr. Robert Webb, USAMRIID) were coated at 4 °C overnight at 1 µg/ml in PBS, then blocked for at least an hour at 37 °C with 4% milk in PBS, 0.1% Tween. After washing, dilution ELISAs were initiated by diluting the VNAs to 25 nM and performing serial dilutions of 1:5. After incubation for one hour at 37 °C, plates were washed and then incubated with 1:10,000 rabbit HRP/anti-E-tag (Bethyl) for one hour, washed, developed with TMB (Sigma) as recommended by manufacturer and read at A450.

Quantitative ELISAs to assess serum VNA levels were performed essentially as described previously^25^. Costar 96-well tissue culture plates were coated with 0.5 mg/ml ciBoNTA, washed and blocked as above. Serum samples from individual mice were diluted 1:1. The internal standard was an E-tagged VHH heterodimer with the same two BoNT/A-neutralizing VHHs as present in each heterohexamer. The standard VNA was diluted to 10 nM in 1:1 untreated mouse sera. All sera and standard were serially diluted 1:2. After 1 h room temperature incubation, the plates were washed and bound VNA detected as above.

### Formulated repRNA preparation

VNA3-ABE amino acid sequence (Fig. S1) was codon optimized and the resulting DNA sequence was synthesized, cloned into a plasmid vector encoding the 5’ and 3’ untranslated regions as well as the nonstructural open reading frame of Venezuelan equine encephalitis virus, strain TC-83, between PflFI and SacII sites, and sequence verified by Twist Biosciences. Plasmid DNA was then digested by NotI restriction enzyme and purified by phenol–chloroform to prepare linear template for RNA production. RNA was then transcribed by T7 polymerase and capped by Vaccinia capping enzyme as previously described^21^. Lithium chloride-precipitated RNA was then resuspended in water and frozen at -80C. Immediately prior to administration, RNA was thawed and mixed in a 1:1 volume with LION at a nitrogen-to-phosphate ratio of 15:1 prior to preparation of syringes for intramuscular administrations. Production of LION was previously described^21^.

### Standard mouse toxin lethality assay

VNA1-ABE and VNA2-ABE were evaluated in vivo using the BoNT murine lethality assay. The lethality assays utilized adult, ~ 20 g female CD-1 mice (Charles River Labs) housed in standard shoebox cages at 5 mice/cage. Following arrival, mice were observed daily and maintained in static cages with standard rodent chow and water ad libitum. One day prior to initiation of each study, mice were weighed and sorted to reduce intergroup weight variation. In studies done at Tufts, VNA1-ABE and VNA2-ABE proteins were co-administered with BoNT via the lateral tail vein in a 200 µl volume and performed as previously described^25^. Each mouse within the respective group received 1 µg of VNA1-ABE (10 pmol) or VNA2-ABE (10 pmol) followed by 100 mouse intraperitoneal median lethal dose (MIPLD_50_) of BoNT/A1, BoNT/B1 or BoNT/E or a combined challenge with 100 MIPLD_50_ each of BoNT/A1, BoNT/B1 and BoNT/E. Monitoring and scoring were as previously described^25^.

For RNA studies, which were performed at Wake Forest, 10 µg of VNA3-ABE RNA or saline formulated in LION were given to mice via bilateral injection into the thigh muscles (5 µg per muscle) 24 h prior to intoxication with 100 MIPLD_50_ each of BoNT/A1, BoNT/B1 and BoNT/E as above. Following treatment or intoxication, mice were observed at least twice daily until study end. During each observation, mice were scored for toxic signs of botulism using the following rubric: (A) respiratory signs: mild abdominal paradox (score of 1), moderate abdominal paradox (3), or severe abdominal paradox and/or agonal respiratory pattern (6); and (B) skeletomuscular signs: lethargy (1), limb weakness (3), or total body paralysis (lack of righting reflex, 6)^36,37^. Animals achieving a score of 6 in either respiratory or neuromuscular categories were immediately euthanized. Mice were given a score of 16 upon death or euthanasia. Time-to-death was recorded at 3 h or in 24 h intervals after intoxication.

### Statistics

Unless otherwise described, continuous variables with normal distribution are presented as mean ± SEM and toxic sign scores are presented as median ± interquartile range (IQR). Toxin potency was determined using simple linear regression. Median time-to-death (MTD) was determined from Kaplan–Meier survival curves and compared among all treatment groups using log-rank test (Mantel-Cox) test. Pairwise comparisons in MTD were only made if significant effects were observed in the full group comparison. Progression of clinical signs was compared among groups using two-way repeated measures ANOVA with Tukey’s multiple comparisons test. Survival outcomes were made using pairwise comparisons against vehicle controls with Fisher’s exact test. Statistical comparisons were made in Prism version 9.0 (Graphpad Software, San Diego, CA). Differences were considered significant at the 95% confidence level (*p* < 0.05).

## Supplementary Information

Supplementary Information.

## Data Availability

Monomer VHH sequences referenced as VA1, VA2, VB1, VB2, VE1, VE2 can be found at GenBank accession numbers: HQ700704.1, HQ700708.1, 6UHT_C, 6UFT_B, ON293151, ON293152, respectively.

## Abbreviations

Ab: Antibody
sdAb: Single-domain antibody
HcAb: Heavy-chain-only antibody
VHH: V_H_ domain of an HcAb
VNA: VHH-based neutralizing agent
BoNT/A: Botulinum neurotoxin serotype A
BoNT/B: Botulinum neurotoxin serotype B
BoNT/E: Botulinum neurotoxin serotype E
HBAT: Heptavalent botulinum antitoxin
MIPLD_50_: Mouse intraperitoneal median lethal dose
PK: Pharmacokinetic
repRNA: Replicating RNA
