# Supplementary material for: Intramuscular delivery of formulated RNA encoding six linked nanobodies is highly protective for exposures to three Botulinum neurotoxin serotypes
Source: Sci Rep. 2022 Jul 8;12:11664. doi: 10.1038/s41598-022-15876-2 (PMC9266081; doi:10.1038/s41598-022-15876-2)
Supplement: Supplementary file 1 — Supplementary Information. [file 41598_2022_15876_MOESM1_ESM.pdf]

INTRAMUSCULAR DELIVERY OF FORMULATED RNA ENCODING SIX LINKED NANOBODIES IS HIGHLY PROTECTIVE FOR EXPOSURES TO THREE BOTULINUM NEUROTOXIN SEROTYPES

SUPPLEMENTARY MATERIALS

1. Supplementary tables

Supplementary table 1. Apparent affinities of mammalian expressed hetermultimeric VNAs for ciBoNTA.

ciBoNTA:

	EC_{50} (nM)	95% CI (nM)
VNA1-ABE	1.03	0.75 – 1.45
VNA2-ABE	1.11	0.75 – 1.77
VNA-BoNT/A	1.45	1.15 – 1.89

ciBoNTB:

	EC_{50} (nM)	95% CI (nM)
VNA1-ABE	1.35	0.99 – 2.04
VNA2-ABE	1.18	0.92 – 1.6
VNA-BoNT/A	0.74	0.47 – 1.4

ciBoNT/E:

	EC_{50} (nM)	95% CI (nM)
VNA1-ABE	0.90	0.63 – 1.38
VNA2-ABE	0.86	0.59 – 1.29
VNA-BoNT/A	0.50	0.40 – 0.63

2. Supplementary Figures

VNA1-ABE

metdtlllwvlllwvpgstgDAAQPARRARRTKLSGAPVPYPDPLEPRAAAGQGVQAQLQLVESGGGLVHPGGSLRLSCAPSASLPSTPFNPFNNMV
GWYRQAPGKQREMVASIGLRINYADSVKGRFTISRDNAKNTVDLQMDSLRPEDSATYYCHIEYTHYWGKGTLVTVSSEPKTPKPQSGGGGSG
GGGSGGGGSGGGGSGGGGSGQGVQAQLQLVESGGGLVQVGGSLRLSCVVSGSDISGIAMGWYRQAPGKRREMVADIFSGGSTDYAGSVKG
RFTISRDNAKKTSYLQMNNVKPEDTGVYYCRLYGSGDYWGQGTQVTVSSAHHSEDPSGGGGQGVQAQLQLVESGGGLVQAGGSLRLSCAAS
ILTYDLDYYYIGWVRQAPGKEREGVSCISSTDGATYYADSVKGRFTISRNNAKNTVYLQMNNLKPEDTAIYYCAAAPLAGRYCPASHEYGYWGQ
GTQVTVSSAHHSEDPSGGGGSGGGGSGGGGSGQGVQAQLQLVESGGGLVQAGGSLRLSCAASEFRAEHFAVGWFRQAPGKEREGVSCVDA
SGDSTAYADSVKGRFTISRDNNKNVVYLQMDSLEPEDTGDYYCGASYFTVCAKSMRKIEYRYWGQGTQVTVSSEPKTPKPQSGGGGQGVQA
QLQLVESGGGLVKPGGSLRLSCVVSGFTFDDYRMAWVRQAPGKELEWVSSIDSWSINTYYEDSVKGRFTISTDNAKNTLYLQMSSLKPEDTAVY
YCAAEDRLGVPTINAHPSKYDYNYWGQGTQVTVSSEPKTPKPQSGGGGSGGGGSGGGGQGVQAQLQLVESGGGLVQAGGSLRLSCAASGR
TFSSYSMGWFRQAPGKEREYVAAVNSNGDSTFYADSIKGRFTVSRDAAKNTVYLQMNSLKPEDTALYYCAAVYGRYTYQSPKSYEYWGQGTQ
VTVSSEPKTPKPQSARQGAPVPYPDPLEPRGGGS*DICLPRWGCLWED**

VNA2-ABE

metdtlllwvlllwvpgstgDAAQPARRARRTKLSGAPVPYPDPLEPRAAAGQGVQAQLQLVESGGGLVHPGGSLRLSCAPSASLPSTPFNPFNNMV
GWYRQAPGKQREMVASIGLRINYADSVKGRFTISRDNAKNTVDLQMDSLRPEDSATYYCHIEYTHYWGKGTLVTVSSEPKTPKPQSGGGGQG
VQAQLQLVESGGGLVQAGGSLRLSCAASILTYDLDYYYIGWVRQAPGKEREGVSCISSTDGATYYADSVKGRFTISRNNAKNTVYLQMNNLKPE
DTAIYYCAAAPLAGRYCPASHEYGYWGQGTQVTVSSAHHSEDPSGGGGQGVQAQLQLVESGGGLVKPGGSLRLSCVVSGFTFDDYRMAWV
RQAPGKELEWVSSIDSWSINTYYEDSVKGRFTISTDNAKNTLYLQMSSLKPEDTAVYYCAAEDRLGVPTINAHPSKYDYNYWGQGTQVTVSSEP
KTPKPQSGGGGQGVQAQLQLVESGGGLVQVGGSLRLSCVVSGSDISGIAMGWYRQAPGKRREMVADIFSGGSTDYAGSVKGRFTISRDNAK
KTSYLQMNNVKPEDTGVYYCRLYGSGDYWGQGTQVTVSSAHHSEDPSGGGGQGVQAQLQLVESGGGLVQAGGSLRLSCAASEFRAEHFAV
GWFRQAPGKEREGVSCVDASGDSTAYADSVKGRFTISRDNNKNVVYLQMDSLEPEDTGDYYCGASYFTVCAKSMRKIEYRYWGQGTQVTVS
SEPKTPKPQSGGGGQGVQAQLQLVESGGGLVQAGGSLRLSCAASGRTFSSYSMGWFRQAPGKEREYVAAVNSNGDSTFYADSIKGRFTVSRD
AAKNTVYLQMNSLKPEDTALYYCAAVYGRYTYQSPKSYEYWGQGTQVTVSSEPKTPKPQSARQGAPVPYPDPLEPRGGGS*DICLPRWGCLWED**

VNA3-ABE

melglswvvlaallqgvqaQLQLVESGGGLVHPGGSLRLSCAPSASLPSTPFNPFNNMVGWYRQAPGKQREMVASIGLRINYADSVKGRFTISRDN
AKNTVDLQMDSLRPEDSATYYCHIEYTHYWGKGTLVTVSSGGGGGQLQLVESGGGLVQAGGSLRLSCAASILTYDLDYYYIGWVRQAPGKERE
GVSCISSTDGATYYADSVKGRFTISRNNAKNTVYLQMNNLKPEDTAIYYCAAAPLAGRYCPASHEYGYWGQGTQVTVSSGGGGGQLQLVESG
GGLVKPGGSLRLSCVVSGFTFDDYRMAWVRQAPGKELEWVSSIDSWSINTYYEDSVKGRFTISTDNAKNTLYLQMSSLKPEDTAVYYCAAEDRL
GVPTINAHPSKYDYNYWGQGTQVTVSSGGGGGQLQLVESGGGLVQVGGSLRLSCVVSGSDISGIAMGWYRQAPGKRREMVADIFSGGSTDY
AGSVKGRFTISRDNAKKTSYLQMNNVKPEDTGVYYCRLYGSGDYWGQGTQVTVSSGGGGGQLQLVESGGGLVQAGGSLRLSCAASEFRAEHF
AVGWFRQAPGKEREGVSCVDASGDSTAYADSVKGRFTISRDNNKNVVYLQMDSLEPEDTGDYYCGASYFTVCAKSMRKIEYRYWGQGTQVT
VSSGGGGGQLQLVESGGGLVQAGGSLRLSCAASGRTFSSYSMGWFRQAPGKEREYVAAVNSNGDSTFYADSIKGRFTVSRDAAKNTVYLQM
NSLKPEDTALYYCAAVYGRYTYQSPKSYEYWGQGTQVTVSSGAPVPYPDPLEPR*

Supplementary figure 1. Sequences of the three heterohexamer VNAs employed in this report. The complete encoded amino acid sequences of the three heterohexamer VNAs; VNA1-ABE, VNA2-ABE and VNA3-ABE, are shown. The amino terminal leader peptide sequences that are removed during the secretion process are displayed in lower case. The E-tag sequences are underlined, and the C-terminal mouse albumin binding peptide (only in VNA1-ABE and VNA2-ABE) is italicized. VNA3-ABE contain the following modifications from VNA2-ABE so as to remove unneeded amino acids: 1) the amino terminal six amino acids of each VHH component (determined by degenerate PCR primers used in VHH-display library construction) was replaced by the alpaca consensus FR1 sequence QVQLVE; 2) E-tag was included only at the C-terminus;

3) the mouse albumin binding peptide was removed; 4) only the ‘core’ VHH sequences considered necessary for binding (**Figure 1B**), and; 5): component VHHs were separated only by a GGGGG spacer.

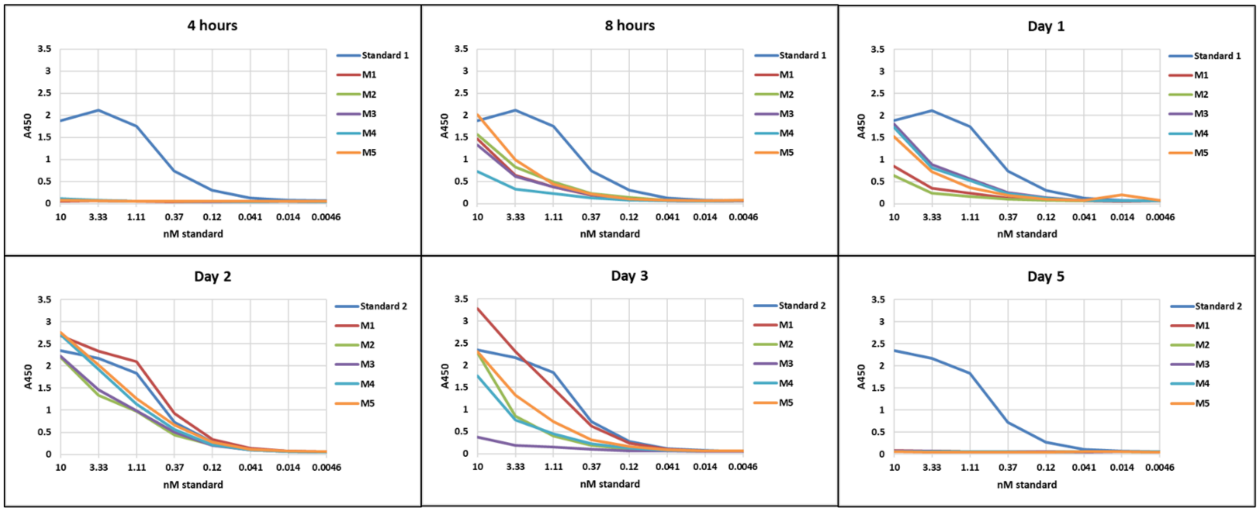

Supplementary figure 2. Serum dilution ELISAs used to assess VNA pharmacokinetics following administration of formulated RNA. ELISAs graphically represents the source data used for Figure 5A, performed as in the Materials and Methods. The EC_{50} values for each sera were estimated and then serum VNA levels established by comparison to the internal standard. This assay was performed twice for statistical evaluation.

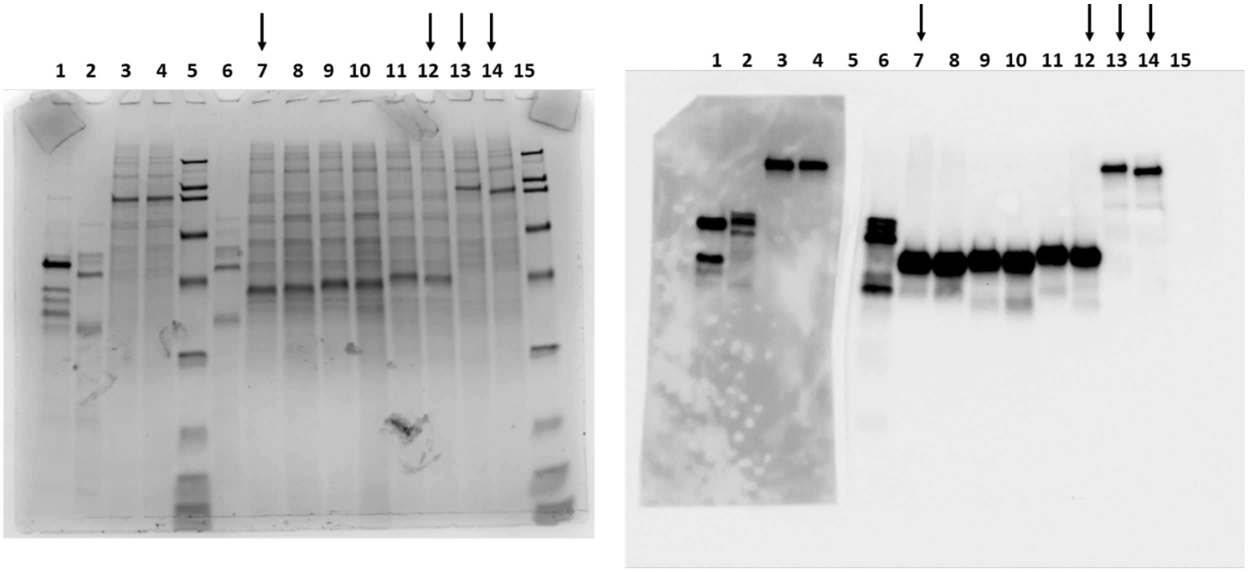

Coomassie stained gel **Western blot**

Supplementary figure 3. Full size original stained gel and western blot used in Figures 2A and 2B. The relevant lanes 7, 12, 13 and 14 that are shown in Figures 2A and 2B are indicated by arrows.
